# Factors associated with Chagas screening among immigrants from an endemic country in Madrid, Spain

**DOI:** 10.1371/journal.pone.0230120

**Published:** 2020-03-13

**Authors:** María Romay-Barja, Teresa Boquete, Obdulia Martinez, Agustin Benito, Teresa Blasco-Hernández

**Affiliations:** 1 Centro Nacional de Medicina Tropical, Instituto de Salud Carlos III, Madrid, Spain; 2 Red de Investigación Colaborativa en Enfermedades Tropicales, RICET, Madrid, Spain; Faculty of Science, Ain Shams University (ASU), EGYPT

## Abstract

**Introduction:**

Approximately 120,000 people live with Chagas disease in Europe, 43% of whom are living in Spain. Early diagnosis and treatment are critical to improve outcomes for those living with Chagas, and also for the prevention of ongoing transmission. The decision to be tested for Chagas is affected by a range of factors. Studies have highlighted the need to consider the wider social determinants of healthcare seeking behaviour related to Chagas. In Madrid, 44% of Bolivians undergo Chagas screening, which is a higher rate than other European regions, but studies concerning the factors which determine testing have not been performed. This study aimed to assess, for a first time, the factors associated with screening for Chagas among Bolivians living in Madrid trying to help in developing strategies and health recommendations.

**Methods:**

This was a cross-sectional survey about knowledge of Chagas and practices of Bolivians living in Madrid, Spain. A structured questionnaire was administered to 376 participants regarding Chagas health-seeking behaviour. Determinants were assessed by multiple logistic regressions adjusted by sex.

**Results:**

After adjusting for others variables and sex, the factors shown to be associated with Chagas screening were to have between 35 and 54 years of age; coming from a department with high prevalence of Chagas (OR 2.17 95% CI 0.99–4.76); received information about Chagas in Spain (OR 2.44 95% CI 1.32–4.51); and received any advice to do the test, especially if the advice came from a professional.

**Conclusions:**

Health authorities should coordinate and promote strategies addressed to diagnose and treat Chagas taking into account all factors associated with screening. Our study suggests that professional advice appears to be the cornerstone to encourage Bolivians to undergo Chagas screening in Madrid. It is time to change the burden of the decision of being screened from the patient to the doctor. Being diagnosed for Chagas needs to become an institutional strategy.

## Introduction

Chagas disease is endemic in 21 Latin American countries where it is estimated that nearly 6 million people are infected [1]. Rural economically disadvantaged and marginalized populations in endemic countries have the highest rates of infection [2]. Endemically transmitted through the faeces of infected triatomine bugs, this neglected tropical disease can also be transmitted through other routes such as vertical transmission, blood transfusion or solid organ transplants [3]. These last routes of transmission have a major role in non-endemic countries where the disease has arrived due to population mobility [4].

Early diagnosis and treatment are critical to improve outcome for those living with Chagas, and also for the prevention of ongoing transmission. Congenital infected new-borns treated within their first year of live have a cure rate of 100% and treating women of reproductive age before being pregnant prevents the vertical transmission of the infection [5]. However, Chronic Chagas disease is considered a disabling disease responsible for most of significant morbidity and mortality due to parasitic diseases[6]. When untreated, Chagas disease remains the leading cause of death from cardiovascular diseases in patients between 30 and 50 years of age in endemic countries [7].

In Europe, it is estimated that 120,000 people live with Chagas disease, of whom approximately 43% are living in Spain [8]. Migrants from Bolivia bear the burden of this parasitic infection, representing 81% of reported cases [9]. Despite substantial increase in Chagas screening in Spain since 2000 [10], late diagnosis and treatment compliance remains a major public health concern in Europe with an estimated of 90% of people living with Chagas unaware of their infection [8]. There is no common European legislation to prevent the transmission of *Trypanosoma cruzi*. The World Health Organization recently recommended implementing active strategies in Europe and other non-endemic regions to detect, screen and diagnose all infected pregnant women as well as their infected newborns and siblings, and to treat them as expeditiously as possible [11].

In Spain, Chagas transmission by blood transfusions and organ transplantation is monitored by law but there is no national strategy regarding vertical transmission or Chagas screening and treatment. Only three regions have an official protocol in which routine testing is recommended in the population coming from endemic areas [12]. In Madrid, a group of health professionals from different institutions have written a guideline encouraging the screening of pregnant women from endemic countries, but adherence to this guideline is poor [13]. Usually, social determinants that foster behavioural changes have been neglected in Chagas disease control strategies [14].

Chagas is considered a silent and poorly visible disease [15]. The decision to undergo testing for Chagas is likely to be affected by a range of factors, including the absence of symptoms in the asymptomatic stage of the infection, accessibility to tests and healthcare, perception of personal risk and prompts to test, such as being offered a test by a healthcare provider [16]. In a non-endemic country, the department of origin and rurality could be a factor related with being screened. People from departments of Bolivia with high prevalence are supposed to be more aware about the disease than people coming from departments where the disease is not endemic.

Other important barriers for testing include fear to a positive result, lack of information about health services [17,18], treatment and working constrains [19]. Studies have emphasized the need to understand and consider the wider social determinants of health-care seeking behaviour related to Chagas into strategies and health recommendations [20].

In Madrid, 44% of Bolivians have undergone Chagas screening [21], which is a higher rate than other European regions, but information concerning factors determining testing is limited. Previous study raised the hypothesis that medical recommendation could be an important factor for Chagas screening [21]. However, the study did not allow for assessing other determinants also associated with having the screening done.

Understanding the socioeconomic determinants of testing patterns among the populations from endemic countries is necessary to guide Chagas screening policies at local and national levels. It is important to establish whether personal determinants were more significant than medical decision in screening of the population at risk. This study is aimed to assess possible factors associated with screening or not for Chagas among Bolivians living in Madrid in an effort to develop strategies and healthcare recommendations.

## Methods

### Study area and population

According to the last municipal census, 15,951 Bolivians are living in Madrid [22]. Most of the Bolivians arrived in Spain between 2002 and 2007, when Bolivian migration flow started to decline due to the EU requirement of a visa followed by the Spanish economic crisis. Most Bolivians live in the southern neighborhoods of Madrid, principally in Usera, Carabanchel, Puente de Vallecas and Latina.

Between 2012 and 2018, a new law mandated universal healthcare available to immigrants with a Public Health Insurance (PHI) card. Access to this card was linked to legal status. Access to healthcare among undocumented immigrants was then reduced to emergency and maternal and child care services [23].

### Sampling and data collection

This cross-sectional study was carried out between March and August 2017 in Madrid, Spain as part of a project aimed at assessing access and use of health services, as well as determining factors, among Bolivians with the diagnosis of Chagas disease in Madrid.

The methodologic aspects have been previously described [21]. A sample size of the Bolivian population living in Madrid was estimated to find that 50% of Bolivians have sufficient knowledge of Chagas disease with a 5% error and 95% confidence level. A total of 376 people were selected among Bolivians attending the waiting room of the Bolivian Consulate in Madrid. A structured questionnaire was administered to participants. Inclusion requirements were age over 18 years and awareness of Chagas disease.

### Data analysis

A descriptive analysis of participants’ characteristics was performed. Differences in sociodemographic characteristics and “being screened or not” were assessed using the chi-squared test for independence for categorical variables. For continuous variables that had a normal or non-normal distribution, we used the Student’s t test or the non-parametric Mann-Whitney test, respectively. *P values* < 0.05 were considered to be statistically significant. Odds ratio (OR) and confidence intervals (CI) were estimated using logistic regression. The variable “Know how Chagas is transmitted” was considered “*yes”* if the respondent mentioned at least one of the possible routes of transmission. The variable “Departments according to Chagas prevalence” considered Cochabamba, Santa Cruz, Chuquisaca and Tarija as the departments of Bolivia where Chagas is endemic, and La Paz, Potosi, Oruro, Beni and Pando as the non-endemic departments.

The collinearity between independent variables was checked and when present, the variable explaining less the data distribution was removed. A multiple logistic regression model adjusted by sex was obtained using a backward stepwise procedure. The OR and 95% CI were computed. P values < 0.05 were considered statistically significant. Data analyses were performed using STATA software (version 15).

### Ethics statement

This study was approved by the Ethics Committee of the Spanish National Health Institute, Carlos III (CEI PI 50_2016). Interviewees signed written informed consent for participation in the study.

## Results

### Descriptive characteristics

The majority of Bolivians living in Madrid were women (57.7%) and had a mean age of 38 years (IQ: 33–45 years, minimum 18 years, maximum 77 years). Nearly 3 out of 4, 74.6% Bolivians said to have finished secondary school or more. Most of the Bolivians interviewed came from Cochabamba (40.7%) or Santa Cruz (39.9%), from urban areas (59.0%) and from a house built with bricks (47.9%); and most of them were married (66.0%) and had children (83%). The majority of the Bolivians who were interviewed held a Public Health Insurance card (86.97%) and related having had no problems to seeing a physician in Spain (75.8%). Bolivians (88.3%) used primary healthcare services when they feel ill but only 166 (44.1%) of Bolivians have Chagas screening performed (S1 Table).

### Chagas’ perceptions and practices

Most of Bolivians living in Madrid knew how Chagas is transmitted (77.93%) that Chagas may be a severe disease (81.91%), and almost half of them that Chagas can’t be cured (49.73%). Also most Bolivians knew that Chagas could be diagnosed and treated in Spain (80.59%). Most of the Bolivians (72.3%) said to have not received information about Chagas in Spain nor advice to have a Chagas test (56.1%), (S2 Table).

### Factors related with diagnosis behaviour

#### Bivariate analysis

Regarding the socio-demographic characteristics, Bolivians between 35 and 54 years of age had > 3 times the odds of having done Chagas screening compared with younger and older Bolivians (Table 1). Regarding their department of origin, the Bolivians coming from a department with high prevalence of Chagas had almost 3 times the odds of having the test for Chagas done. Other socio-demographic characteristics such as the level of education, year of arrival, current job, household income, and having a PHI card did not show an association with Chagas’ diagnosis behaviour among the Bolivians living in Madrid.

**Table 1:** Factors associated with having done the Chagas' test by multiple logistic regression.

	Unadjusted OR (95% CI)			Adjusted OR (95% CI)		
**Age**						
18–24	1					
25–34	1.95	(0.72-	5.26)	3.21	0.65	15.99
35–44	4.23	(1.62-	11.00)	8.71	1.79	42.46
45–54	3.94	(1.44-	10.81)	11.35	2.23	57.72
55–64	3.51	(1.04-	11.84)	6.44	0.99	41.88
> 65	2.40	(0.57-	10.05)	5.90	0.69	50.11
**Children**						
Yes	1					
No	1.78	(1.01-	3.14)			
**Departments according to Chagas prevalence**						
Low prevalence	1					
High prevalence	2.96	(1.56-	5.61)	2.17	0.99	4.76
**Know how Chagas is transmitted**						
No	1					
Yes	2.47	(1.48-	4.11)			
**Have you ever seen a Vinchuca?**						
No	1					
Yes	1.71	(1.07-	2.72)			
**Do you think Chagas is a severe disease?**						
No	1					
Yes	1.84	(1.05-	3.20)			
**Chagas disease heals?**						
Yes	1					
No	0.93	(0.59-	1.48)			
Don't know	0.49	(0.27-	0.91)			
**Have you received information about Chagas in Spain?**						
No						
Yes	4.01	(2.47-	6.49)	2.44	1.32	4.51
**It is possible to do the test of Chagas in Spain?**						
Yes						
No	1.68	0.28	10.19			
Don't know	0.47	0.26	0.82			
**Do you know if Chagas could be treated in Spain?**						
Yes						
No	0.51	0.30	0.89			
**Have you ever received advice to do the test?**						
No						
Yes, from a friend	2.27	(0.99-	5.22)	1.57	0.65	3.78
Yes, from a relative	2.91	(1.51-	5.61)	2.12	1.04	4.35
Yes, from a NGO	9.92	(1.94-	50.72)	7.31	1.32	40.48
Yes, from a doctor	61.71	(18.50-	205.85)	60.70	16.98	217.02
**Do you know someone positive to Chagas?**						
No						
Yes	1.90	(1.21-	2.97)			

According to the bivariate analysis; knowing at least one route of Chagas transmission, thinking that Chagas is a severe disease; having received information about Chagas in Spain and knowing someone who had Chagas, have a positive association with having a Chagas test performed. Conversely, not knowing if the disease can be cured, not knowing if it is possible to be tested and not knowing if is possible to be treated in Spain, have around 2 times the odds of did not have the Chagas test performed.

Finally, receiving any advice to be screened has 8.06 times the odds (p-0.000) of having the Chagas test done, and particularly associated if such advice comes from a physician.

While they had no difficulty with being seen by a physician; the place where they seek treatment when they feel ill and thinking that the physicians in Spain are familiar with Chagas disease, were factors which shown no association with having the Chagas screening performed.

#### Multivariate analysis

Once adjusted for others variables and sex, Bolivians between 35 and 54 years of age were four times more likely to have been screened for Chagas than those younger or older (Table 1). Bolivians coming from a department with high prevalence of Chagas were twice more likely to have been screened than those coming from departments of low Chagas prevalence, but this association was not significant. The Bolivians who had received information about Chagas in Spain had 2.4 fold increase probability of having the Chagas’ test performed than those who had not received any information. Also the Bolivians who had received advice to do the test from a relative had 2.1 fold increased probability of having Chagas screening than those who did not receive advice and 7.3 if the advice came from a NGO. Finally, if the advice came from a physician, the probability of having the screening done was up 10 times higher.

## Discussion

This study highlights several factors associated with the Chagas testing behaviour among Bolivians living in Madrid. These factors could have important public health implications in the design of Chagas screening initiatives addressed to increase the screening of the population at risk in a non-endemic country. Our study suggests that Bolivians who said to “don’t know” if the disease can be cured or not and “don’t know” if it is possible to be tested and treated in Spain, were less likely to have their Chagas screening done. While Bolivians of middle age, aware about Chagas severity, coming from an endemic department and who have received information about Chagas in Spain, were more likely to have the Chagas screened done. Finally, the main factors associated with having Chagas screened were having received information about Chagas in Spain and having received advice to do the test, especially if that advice comes from a professional, from an NGO or a physician.

Only 44% of Bolivians living in Madrid have been tested for Chagas and most of them were between 35 and 54 years of age. In Madrid, women have been the target population for Chagas interventions [24], but no differences in screening based on sex were found. The existence of a professional consensus document addressed to screening women appears to have had little impact on the population at risk. Moreover, given the importance of screening all Bolivian women of childbearing age to prevent vertical transmission [5], a clear effort should be made in Madrid to improve the rate of screening among younger female Bolivian population, preferably before becoming pregnant.

People from endemic areas do not always perceived Chagas disease as a threat [25,26]. In our study, coming from a rural area or from a house of adobe did not have a significant association with being screened for Chagas, while coming from an endemic department is a determinant of having the screening test done, despite the displacements from endemic rural areas to urban settings and from endemic departments to non-endemic departments [27]. Only Bolivians from La Paz, where Chagas is not endemic, were less likely to have a diagnostic test performed. However, those who resided in an urban area whose parents migrated from an endemic department need to be aware of the risk of vertical transmission and, in Spain, all Bolivians should be included in any screening strategy [28].

Other socioeconomic characteristics related to Bolivians living in Madrid, such as low income, type of job or not having a PHI card were not determinants for screening. Poor access to health services and irregular status are known barriers for immigrants to undergo screening for Chagas in non-endemic countries [19,29,30] but most of the Bolivian population currently living in Madrid arrived more than 12 years ago, have a stable status and have access to public health services. However, even when most of Bolivians knew that is possible to be tested and treated in Madrid, answered “don’t know” was associated with not doing the Chagas screening. Also, Bolivians who did not know if the disease can be cured were less likely to have their Chagas screening done, even more that those who said that the Chagas has no cure.

While many studies have highlighted that increasing knowledge about Chagas disease is not always enough to change care-seeking behaviour [31,32], others studies have shown that being aware of the disease is an important factor for being screened [17]. Our study found that people who knew how Chagas is transmitted, those who have seen Vinchucas and those who knew someone with Chagas, were more likely to do their Chagas screening. Additionally, to have received information about Chagas in Spain is a clear determinant to be tested. Previous studies have shown that experiences and misconceptions from the country of origin are important barriers that explain the reluctance of immigrants to undergo screening when first arriving in Spain [33]. After a decade of living in Madrid, having received some information in Spain about the disease was an important factor to perform the test for Chagas. Since 2012 some public-private partnerships have been established in Madrid and different non-profit organizations have carried out some educational campaigns [34]. Reinforcing such educational campaigns would help clarify former misconceptions about the disease and promote screening. Once again, emphasis is needed in women in reproductive age and children if the objective is to accelerate the elimination of congenital transmission of Chagas in European non-endemic countries [11].

Because most Bolivians utilize the Health Centers, routine targeted Chagas screening programs for diagnosis and treatment would be more efficient if applied in primary healthcare services. An official protocol for Chagas screening at primary healthcare services would help reduce barriers to Chagas screening and treatment and would improve the follow-up of the patients. However, the lack of awareness among physicians at primary healthcare of the risk of Chagas in Madrid [35] has to be solved.

Finally, receiving advice to undergo testing for Chagas was the main factor associated Bolivians who have received advice are from 2 to 60 times more likely of having the screening done than those who have not received advice especially if such advice came from an NGO or a physician. However, doctors at primary healthcare services used to wait until the patient request the test to prescribe a Chagas test [35]. Being diagnosed for Chagas needs to become an institutional strategy instead of a personal decision [13], especially when it is known that in Spain it would be cost-efficient to perform screening programs on the general Bolivian population [28].

This study had some limitations. First, it was a cross-sectional study conducted in Madrid, so the findings might not be generalized to very different contexts. Second, recall accuracy about their screening and their results could be a problem, especially if the test was negative. Third, due to sample size, some associations may not show significance in multivariable logistic regression.

## Conclusions

Health authorities should coordinate and promote strategies addressed to diagnose and treat Chagas that take into account all factors found associated with screening. Our study suggests that professional advice, coming from an NGO or a physician, is the cornerstone to encourage Bolivians to undergo Chagas screening. To reinforce the screening of Chagas to younger population, especially women, seems necessary if the aim is to end with vertical transmission. Being diagnosed for Chagas has to become an institutional strategy. Bolivians living in Madrid should be offered serologic testing as part as their routine health care regardless of their department of origin, occupation or economic status. Spain, being the country with most cases of Chagas in Europe, should be leading this sort of approaches.

## Supporting information

S1 Table Socioeconomic characteristics of participants by being screened or not. (PDF)

S2 Table Chagas knowledge and practices by being screened or not. (PDF)
