# Supplementary material for: Factors associated with Chagas screening among immigrants from an endemic country in Madrid, Spain
Source: PLoS One. 2020 Mar 13;15(3):e0230120. doi: 10.1371/journal.pone.0230120 (PMC7069611; doi:10.1371/journal.pone.0230120)
Supplement: S1 Table — (PDF) [file pone.0230120.s001.pdf]

S1 Table 1. Socioeconmic characteristics of participants by being screened or not

	Test not done		Test done		*P-value*
**Sex**	**n=210**	**%**	**n=166**	**%**	
Female	114	54.29	103	62.05	
Male	96	45.71	63	37.95	0.130
**Age**					
18-24	23	10.95	6	3.61	
25-34	63	30.00	32	19.28	
35-44	68	32.38	75	45.18	
45-54	36	17.14	37	22.29	
55-64	12	5.71	11	6.63	
> 65	8	3.81	5	3.01	0.006
**Marital status**					
Partner	63	30.00	38	22.89	
Married	73	34.76	74	44.58	
Single	53	25.24	40	24.10	
Widow	8	3.81	3	1.81	
Divorced	13	6.19	11	6.63	0.251
**Education**					
Primary school or less	49	23.33	48	28.92	
Secondary school or more	161	76.67	118	71.08	0.163
**Children**					
Yes	167	79.52	145	87.35	
No	43	20.48	21	12.65	0.045
**Year of arrival**					
< 2000	5	2.38	3	1.81	
2000 - 2005	116	55.24	84	50.60	
2006 - 2010	66	31.43	61	36.75	
2011 - 2017	23	10.95	18	10.84	0.730
**Bolivian department**					
Cochabamba*	79	37.62	74	44.58	
Santa Cruz*	80	38.10	70	42.17	
La Paz	27	12.86	8	4.82	
Potosi	6	2.86	3	1.81	
Chuquisaca*	3	1.43	6	3.61	
Oruro	6	2.86	1	0.60	
Beni	5	2.38	2	1.20	
Tarija*	3	1.43	2	1.20	
Pando	1	0.48	0	0.00	0.076
**Departments according to Chagas prevalence**					
Endemic department	45	21.43	14	8.43	
Non-endemic department	165	78.57	152	91.57	0.001
**Area**					
Rural	55	26.19	45	27.11	
Urbano	155	73.81	212	127.71	0.841
**House material in Bolivia**					
Adobe	44	20.95	45	27.11	
Brick	108	51.43	72	43.37	
Adobe & brick	30	14.29	26	15.66	
Brick & concrete block	16	7.62	14	8.43	
Concrete block	12	5.71	9	5.42	0.566
**Madrid District**					

Less than 10% of Bolivian population	74	35.24	58	34.94	
More than 10% of Bolivian population	136	64.76	108	65.06	0.952
**Public health card**					
No	29	13.81	20	12.05	
Yes	181	86.19	146	87.95	0.614
**Are you currently working?**					
No	72	34.29	47	28.31	
Yes	138	65.71	119	71.69	0.216
**Jobs**					
Professionals, managers & technicians	6	2.86	6	3.61	
Services & sales	41	19.52	31	18.67	
Manual jobs	91	43.33	84	50.60	0.758
**Household income**					
No one	7	3.45	5	3.13	
<1000 €	80	39.41	74	46.25	
1001-2000 e	101	49.75	66	41.25	
>2000 €	15	7.39	15	9.38	
Don't know	7	3.45	6	3.75	0.580

*Departments where Chagas is endemic in Bolivia
