# Supplementary material for: Factors associated with Chagas screening among immigrants from an endemic country in Madrid, Spain
Source: PLoS One. 2020 Mar 13;15(3):e0230120. doi: 10.1371/journal.pone.0230120 (PMC7069611; doi:10.1371/journal.pone.0230120)
Supplement: S2 Table — (PDF) [file pone.0230120.s002.pdf]

**S2 Table2. Chagas knowledge and practices by being screened or not**

| | Test not done | | Test done | | *P-value* |
|---|---|---|---|---|---|
| | n | % | n | % | |
| **To know how Chagas is transmited** | | | | | |
| No | 66 | 31.43 | 26 | 15.66 | |
| Yes | 144 | 68.57 | 149 | 89.76 | 0.000 |
| **Have you ever seen a Vinchuca?** | | | | | |
| No | 69 | 32.86 | 37 | 22.29 | |
| Yes | 141 | 67.14 | 129 | 77.71 | 0.024 |
| **Do you think Chagas is a severe disease?** | | | | | |
| No | 46 | 21.90 | 22 | 13.25 | |
| Yes | 164 | 78.10 | 144 | 86.75 | 0.032 |
| **Chagas disease can be cured?** | | | | | |
| Yes | 60 | 28.57 | 56 | 33.73 | |
| No | 100 | 47.62 | 87 | 52.41 | |
| Don't know | 50 | 23.81 | 23 | 13.86 | 0.051 |
| **Have you received information about Chagas in Spain?** | | | | | |
| No | 177 | 84.29 | 95 | 57.23 | |
| Yes | 33 | 15.71 | 71 | 42.77 | 0.000 |
| **If you feel ill, where you go?** | | | | | |
| Health center | 187 | 89.05 | 145 | 87.35 | 0.661 |
| Hospital emergency room | 19 | 9.05 | 26 | 15.66 | 0.050 |
| Self-medicate | 24 | 11.43 | 9 | 5.42 | 0.041 |
| Pharmacy | 2 | 0.95 | 0 | 0.00 | 0.207 |
| **Have you ever had problems to go to the doctor in Spain?** | | | | | |
| No | 163 | 77.62 | 122 | 73.49 | |
| Yes | 47 | 22.38 | 44 | 26.51 | 0.354 |
| **Do you think that Spanish doctors in health centers know Chagas?** | | | | | |
| No | 82 | 39.05 | 65 | 39.16 | |
| Yes | 112 | 53.33 | 91 | 54.82 | |
| Don't know | 16 | 7.62 | 10 | 6.02 | 0.827 |
| **It is possible to do the test of Chagas in Spain?** | | | | | |
| Yes | 160 | 76.19 | 143 | 86.14 | |
| No | 2 | 0.95 | 3 | 1.81 | |
| Don't know | 48 | 22.86 | 20 | 12.05 | 0.022 |
| **Do you know if Chagas could be treated in Spain?** | | | | | |
| Yes | 160 | 76.19 | 143 | 86.14 | |
| No | 50 | 23.81 | 23 | 13.86 | 0.015 |
| **Have you ever received advice to do the test?** | | | | | |
| No | 162 | 77.14 | 49 | 29.52 | |
| Yes | 48 | 22.86 | 117 | 70.48 | 0.000 |
| **If yes, from who?** | | | | | |
| A friend | 16 | 33.33 | 10 | 8.55 | 0.545 |
| A familiar | 26 | 54.17 | 22 | 18.80 | 0.801 |
| NGO | 1 | 2.08 | 6 | 5.13 | 0.025 |
| A doctor | 3 | 6.25 | 56 | 47.86 | 0.000 |
| **Do you know someone positive to Chagas?** | | | | | |
| No | 104 | 49.52 | 46 | 38.66 | |
| Yes | 106 | 50.48 | 120 | 100.84 | 0.000 |
